# Moonlighting Proteins and Cardiopathy in the Spatial Response of MCF‐7 Breast Cancer Cells to Tamoxifen

**DOI:** 10.1002/prca.201900029

**Published:** 2019-07-25

**Authors:** Abdulrab Ahmed M. Alkhanjaf, Roberto Raggiaschi, Mark Crawford, Gabriella Pinto, Jasminka Godovac‐Zimmermann

**Affiliations:** ^1^ Proteomics and Molecular Cell Dynamics Division of Medicine School of Life and Medical Sciences University College London NW3 2PF London UK; ^2^ Molecular Biotechnology, Department of Clinical Laboratory Sciences College of Applied Medical sciences Najran University Najran 61441 Saudi Arabia; ^3^ Department of Chemical Sciences University of Naples Federico II 80126 Naples Italy

**Keywords:** mass spectrometry, MCF‐7 cells, SILAC‐based quantitative proteomics, subcellular protein location, tamoxifen

## Abstract

**Background:**

The purpose of this study is to apply quantitative high‐throughput proteomics methods to investigate dynamic aspects of protein changes in nucleocytoplasmic distribution of proteins and of total protein abundance for MCF‐7 cells exposed to tamoxifen (Tam) in order to reveal the agonistic and antagonistic roles of the drug.

**Experimental design:**

The MS‐based global quantitative proteomics with the analysis of fractions enriched in target subcellular locations is applied to measure the changes in total abundance and in the compartmental abundance/distribution between the nucleus and cytoplasm for several thousand proteins differentially expressed in MCF‐7 cells in response to Tam stimulation.

**Results:**

The response of MCF‐7 cells to the Tam treatment shows significant changes in subcellular abundance rather than in their total abundance. The bioinformatics study reveals the relevance of moonlighting proteins and numerous pathways involved in Tam response of MCF‐7 including some of which may explain the agonistic and antagonistic roles of the drug.

**Conclusions:**

The results indicate possible protective role of Tam against cardiovascular diseases as well as its involvement in G‐protein coupled receptors pathways that enhance breast tissue proliferation.

## 1 Introduction

Hormone receptor positive (HR+) breast cancers represent 70% of all breast tumors. Their oncogenesis is a multiple step process thought to be driven by two transcription factors, the estrogen receptor (ER) and/or the progesterone receptor (PR).^1^ Consequently, the endocrine‐targeted therapeutic approach has focused on both receptors as prognostic markers and therapeutic targets,^2^ aiming to alter the estrogen signaling for patients with ERα‐positive disease.^3^

Over the past four decades tamoxifen (Tam) has been extensively used in neoadjuvant and adjuvant settings for the treatment of hormonal dependent breast cancers by acting as a competitive inhibitor of ER.^3^, ^4^, ^5^ The benefits gained from the treatment are limited by development of either de novo or acquired resistance following a period of response to Tam. For both types of resistance, the lack of pathological response to Tam is manifested when the tumor cells commence proliferation, challenging the clinical management of patients.^6^

The ER mediated signaling pathways involve genomic, non‐genomic, or mitochondrial pathways that contribute to amplification of the multistep process of tumor development.^7^ It is well established that binding of estrogen (E2) to ERs leads to subsequent receptor dimerization and recruitment of other coactivators such as SRC‐3 and transcription factors such as AP‐1. This complex acts directly as a transcriptional factor by binding to estrogen responsive elements (EREs) that are located on the promotor regions of the target genes. This initiates transcription of the target genes leading to proliferation, apoptotic inhibition, and uncontrolled growth.^8^ Full agonistic activity of E2 is achieved via the activation of two transactivation domains known as activation function 1 (AF‐1), regulated by growth factors, and activation function 2 (AF‐2), regulated by E2 binding.^5^ Tam acts as a selective modulator with partial agonistic activity in which the active metabolite of Tam, 4‐hydroxytamoxifen (4‐OHT), binds to a ligand‐binding domain (LDB) of ER. This promotes conformational changes of the ER dimers, leading to recruitment of transcriptional repressors that relocate helix 12 and prevent the coactivator binding and activation of AF‐2. Subsequently, the transcriptions of AF‐2 dependent genes are downregulated, but partial agonistic activities can be initiated for the AF‐1 dependent genes.^8^ Consequently, Tam can inhibit the transcriptional activities of ER in some tissues such as the breast tissue but activate transcriptional cascades in other tissues such as uterus. Therefore, Tam and related compounds show mixed agonist/antagonist properties and are classified as estrogen selective modulators.

In breast tissue Tam acts predominantly as an antagonist but there is some evidence that Tam agonistic activities can enhance breast tissue proliferation, thereby leading to limited efficacy and resistance.^9^ Furthermore, at the molecular level the active metabolite of Tam, 4‐OHT, can cause the induction of gene expression related to the cell cycle, particularly for those genes induced by E2.^10^ Numerous studies have tackled anti‐estrogen resistance therapy using different approaches. These include various cell line models in culture and/or as xenografts as well as in studies in the clinical adjuvant and/or neoadjuvant settings.^6^ Owing to the complexity and heterogeneity of breast cancer, understanding the exact mechanisms underlying resistance to Tam remains far from being elucidated. The proposed mechanisms are diverse and consistently indicate that complex cellular mechanisms are involved in the developing resistance. Studies related to the differential metabolism of Tam, mutated/downregulated ER, cross‐talk between ER and growth factor transduction pathways, dynamic changes in cell signaling, and cellular responses to oxidative stress have all been carried out, with limited explicative success.^3^, ^11^ These studies suggest that numerous pathways are intertwined and that more integrative approaches using high‐throughput technologies that monitor large numbers of proteins in parallel are required to elucidate Tam function, to predict and overcome resistance, and to ultimately lead to better therapeutic interventions.

We have developed high‐throughput proteomics methods suitable for investigating dynamic aspects of protein subcellular distribution in the response of cells to stimulations.^12^, ^13^, ^14^, ^15^, ^16^ This approach, which combines global quantitative proteomics with the analysis of fractions enriched in target subcellular locations, has allowed measurement of the changes in total abundance and in the compartmental abundance/distribution between the nucleus and cytoplasm for several thousand proteins differentially expressed in MCF‐7 cells in response to estrogen stimulation. Using stable isotope labelling in cell culture (SILAC) and quantitative high‐resolution mass spectrometry (MS) analysis demonstrated that estradiol stimulation of MCF‐7 cells results in strong redistribution of massive numbers of proteins between the nucleus and cytoplasm. Many more proteins showed appreciable spatial changes in compartmental abundance than in total protein abundance.^14^ We suggested^14^, ^15^ that major alterations in the spatiotemporal subcellular distribution of proteins are the dominant response of MCF‐7 cells to estradiol exposure, that a major role of the estrogen receptor and possibly other nuclear hormone receptors may be the “polling” of and response to spatially distributed functional networks, and that strong perturbation of subcellular spatial regulation may be a crucial feature of breast cancer. In the present work, we apply global, quantitative proteomics analysis of changes in nucleocytoplasmic distribution of proteins and total protein abundance for MCF‐7 cells exposed to Tam.

## 2 Experimental Section

### 2.1 Cell Culture and SILAC Labeling

MCF‐7 (Michigan Cancer Foundation) cells were purchased from the ATCC (HTB‐22, Manassas, VA) and cultured in a humidified incubator at 37 °C with 5% CO_2_, the cells were maintained in DMEM/F‐12 (1:1) (Ham) 1× with l‐glutamine, 15 mm HEPES supplemented with 10% fetal bovine serum (FBS), and 1% antibiotic–antimycotic (10 000 units penicillin, 10 mg streptomycin, and 25 µg Amphotericin B per milliliter).

**Clinical Relevance**

We present large‐scale quantitative subcellular proteomics analysis of MCF‐7 breast cancer cells stimulated by 4‐hydroxytamoxifen (4‐OHT). The response of MCF‐7 cells to the tamoxifen (Tam) treatment shows significant changes in subcellular distribution rather than in their total abundance. Extensive bioinformatics analysis suggests that cellular spatial reorganization is a major component of the molecular basis of Tam function and its use in breast cancer therapy. The study suggests numerous pathways involved in 4‐OHT response of MCF‐7 including some of which may explain the agonistic and antagonistic role of the drug.

MCF‐7 cells were adapted to grow in DMEM:F12 medium, the cells were split into two different populations and seeded into two culture flasks: one containing non‐radioactive isotopic labeled heavy amino acids (^13^C_6_ l‐lysine‐2HCl and ^13^C_6_ 15N4 l‐arginine‐HCl) and the second with normal amino acids (l‐lysine‐2HCl and l‐arginine‐HCl) SILAC media. Both cell populations were grown at 37 °C in a humidified incubator of 5% CO_2_ and were passaged for at least five cell doublings by splitting cells at 70–80% confluence. After passage five, high incorporation efficiency of heavy amino acids, (^13^C_6_ l‐lysine‐2HCl and 13C6 15N4 l‐arginine‐HCl) was verified (>95%) by MS analysis of peptides from heavy‐ and light‐labeled cells. Both cell populations (light and heavy) were then expanded to the number required for subsequent fractionation.

### 2.2 Cell Survival/Cytotoxicity Assay

A 200 µm stock solution of 4‐OHT was prepared in absolute ethanol and stored at −20 °C. Cells were plated at density of 12.5 × 10^3^ cells by adding 100 µL in a 96‐well plate and incubated for 24 h at 37 °C in a humidified incubator of 5% CO_2_. Upon confluence, media were changed into DMEM‐F12 phenol red free medium supplemented with 10% charcoal‐treated FBS and the medium was changed every 24 h before 4‐OHT stimulation. At 48 h, cells were treated with various concentrations of 4‐OHT (0.5, 0.1, 1.5, 2.0 µm) in three replicates for each dose. With each dose, a negative control was plated with the same cell number and treated with an equivalent volume of 0.1% ethanol (maximum 0.1%). After 24 h from the stimulation time point, 10 µL of cell counting kit‐8 (one tenth of the cells plus media) was added to each well including the negative control and blank wells. The plate was placed back in a CO_2_ incubator for 3.5 h. Colorimetric readings were taken on a microplate reader using a filter for 450 nm.

### 2.3 Hydroxytamoxifen (4‐OHT) Treatment of MS Samples

To treat the desired number of cells with 1 µm 4‐OHT, the SILAC media were changed into the phenol red free MEM for SILAC (Thermo Scientific, UK) supplemented with 10% dialyzed FBS and 1% of antibiotic–antimycotic solution and incubated for 24 h at 37 °C in a humidified incubator of 5% CO_2_. Both cell populations were subjected to a further 24 h (total of 48 h) incubation in a fresh phenol red free MEM for SILAC. After 48 h, a dose of 1 µm 4‐OHT in fresh phenol red free MEM for SILAC was applied for 24 h to the heavy cell population, while the media was changed into a fresh phenol red free MEM for SILAC (without treatment) in the light‐labeled cell population.

### 2.4 Subcellular Fractionation and Enrichment

Differential centrifugation was used to obtain the nuclear, cytoplasmic, and total lysate fractions accordingly using the protocol previously described.^14^, ^17^ All the subsequent steps were carried out at 4 °C with all buffers containing protease and phosphatase inhibitors (Roche Diagnostics, Mannheim, Germany). Light‐ and heavy‐labeled cell populations grown as monolayers in T75 flasks were washed in ice‐cold phosphate buffer saline (PBS) pH 7.4 three times, cells were scraped from culture flasks on ice using a plastic cell scraper and collected in 15 mL conical centrifuge tubes.

For a total lysate, 12 × 10^6^ cells were scraped from heavy and light cell populations in ice‐cold PBS and the pellet was collected by cold centrifugation at 300 × *g* for 5 min (Mistral 3000i Refrigerated Centrifuge, 4312–708 BS 4402 rotor). Two pellets were collected, each pellet was dissolved and resuspended in cold RIPA lysis buffer (50 mm Tris‐HCl at pH 7.5, 300 mm NaCl, 1% NP40, 0.5% sodium deoxycholate, 0.1% SDS, 1 mm EDTA), transferred to a pre‐chilled 1.5 mL microcentrifuge tube, the mixture was agitated on ice for 15–30 min (vortexed every 5 min) and centrifuged at 300 × *g* for 5 min (Heraeus Biofuge Pico, Thermo Fisher Scientific, UK). The cell debris was pelleted by cold centrifugation at 300 × *g* for 5 min (Heraeus Biofuge Pico, Thermo Fisher Scientific, UK), and the supernatant was collected as total lysate (T). For nuclear and cytoplasmic fractions the cells were counted and 12 × 10^6^ cells from light and heavy cell populations were recovered from the culture flasks as described in the previous section. The pellets were obtained by cold centrifugation at 300 × *g* for 5 min (Mistral 3000i Refrigerated Centrifuge, 4312–708 BS 4402 rotor). Each pellet was allowed to stand on ice for 10 min in a hypotonic osmotic buffer (10 mm NaCl, 1.5 mm MgCl_2_, 10 mm Tris‐HCl at pH 7.4) to swell the cell membrane of the cells, the cells were pelleted and the supernatant was removed and in a subsequent step centrifuged at 300 × *g* (Mistral 3000i Refrigerated Centrifuge, 4312–708 BS 4402 rotor). Each pellet was resuspended in ice‐cold isotonic sucrose (breaking) buffer containing (300 mm sucrose, 1 mm EDTA, heparin 5 U mL^−1^, 10 mm HEPES, 5 mm MgCl_2_ at pH 7.4), the cells were homogenized by 10–25 strokes of the pestle of a tight‐fitting Dounce homogenizer (0.05–0.08 clearance). Under phase contract microscope, the suspension was inspected after each ten strokes and homogenization was continued until about 90% of cells have been broken. The obtained lysate that contained the subcellular homogenate was subjected to a cold centrifugation at 800 × *g* for 10 min to separate the nuclear pellet (N) from the crude cytoplasmic supernatant (C). The supernatant was collected and labelled as a cytoplasmic fraction (C).

The nuclear pellet (N) was suspended in a hypotonic buffer (10 mm HEPES at pH 7.9, 10 mm KCl, 5 mm MgCl_2_, 2 mm EDTA, 1 mm dithiothreitol [DTT], 0.1% Triton X‐100), and incubated for 15 min at 4 °C on an end‐over‐end rotator. To release the nuclear proteins, the nuclei were pelleted and the pellet was suspended in high salt breaking buffer containing (20 mm HEPES at pH 7.9, 700 mm NaCl, 1.5 mm MgCl_2_, 1 mm EDTA, 10% glycerol), for 2 h at 4 °C on an end‐over‐end rotator. The supernatant was collected as nuclear‐enriched fraction (N) and separated from the pelleted nuclear debris by centrifugation of the high salt extracts for 10 min at 800 × *g* (Heraeus Biofuge Pico, Thermo Fisher Scientific, UK) and labeled as nuclear fraction (N).

Both the nuclear fraction (N) and cytoplasmic (nucleus‐depleted) supernatant (C) were subjected to acetone precipitation by adding four volumes of 80% acetone at −20 °C for 1 h, the pellets were precipitated by further cold centrifugation step at 16 000 × *g* (Heraeus Biofuge Pico, Thermo Fisher Scientific, UK) and left to dry. The dried pellets were solubilized in a 1× protein solubilization buffer (20 mm PIPES at pH 7.3, 300 mm NaCl, 2% Triton X‐100, 0.2% SDS, 2% sodium deoxycholate). The protein concentration, in each fraction, was quantified using a BCA protein assay kit (The Thermo Scientific Pierce, Rockford, IL) by measuring the absorbance of protein samples at 562 nm.

### 2.5 Mass Spectrometry Sample Preparation and In‐Gel Digestion

The solubilized protein concentration was measured for the cytoplasmic, nuclear, and total lysate sample types (C, N, T) obtained from the SILAC labelled (heavy) and unlabeled (light) cell populations. Both labelled and unlabeled protein extracts were mixed in 1:1 ratio for each sample type and the proteins in the complex samples were separated based on their molecular weight using 4–15% SDS‐PAGE. The separated protein bands were visualized by silver staining (ProteoSilver Plus, Sigma Aldrich, Poole, UK) and the bands were excised (27–30 horizontal slices per lane) from the gel lane. Each band was cut into 1 mm cubes, placed in a 96‐well plate, and incubated at RT for 10 min in 100 µL ddH_2_O.

Silver stain was removed by immersion of the bands in 100 µL of a destaining solution (100 mm sodium thiosulfate and 30 mm potassium ferricyanide solutions mixed in a 1:1 v:v ratio) at RT for 10 min. After several washes with ddH_2_O to remove residual destaining solution, cycles of dehydration (100% acetonitrile), rehydration (25 mm ammonium bicarbonate), reduction (10 mm DTT, and alkylation (100 mm iodoacetamide) for 5 h in the ProGest Investigator Instrument (DigiLab, Genomics Solutions, Cambs, UK). Upon completion of these cycles, Trypsin Gold, Mass Spectrometry Grade (Promega, Madison, USA) in 50 mm ammonium bicarbonate was added in each well containing dried gel pieces and incubated overnight at 37 °C. Next day, 0.1% formic acid was added to stop the trypsinolysis and the eluted tryptic peptides were collected in MS glass vials, vacuum dried, and dissolved in 0.1% formic acid for LC‐MS/MS.

### 2.6 Mass Spectrometry Analysis

LC‐MS/MS analysis was performed with an Orbitrap/LTQ‐Velos mass spectrometer (Thermo Fisher Scientific, UK). Peptide samples were loaded using a Nanoacquity UPLC (Waters, UK) with Symmetry C18 180 um × 20 mm (Waters part number 186 006 527) trapping column for desalting and then introduced into the MS via a fused silica capillary column (100 µm i.d.; 360 µm o.d.; 15 cm length; 5 µm C18 particles, Nikkyo Technos CO, Tokyo, Japan) and a nanoelectrospray ion source at a flow rate at 0.42 µL min^−1^. The mobile phase comprised H_2_O with 0.1% formic acid (Buffer A) and 100% acetonitrile with 0.1% formic acid (Buffer B). The gradient ranged from 1% to 30% buffer B in 95 min followed by 30% to 60% B in 15 min and a step gradient to 80% B for 5 min with a flow of 0.42 µL min^−1^. The full scan precursor MS spectra (400–1600 *m*/*z*) were acquired in the Velos‐Orbitrap analyzer with a resolution of *r* = 60 000. This was followed by data dependent MS/MS fragmentation in centroid mode of the most intense ion from the survey scan using collision induced dissociation (CID) in the linear ion trap: normalized collision energy 35%, activation Q 0.25; electrospray voltage 1.4 kV; capillary temperature 200 °C isolation width 2.00. The targeted ions were dynamically excluded for 30 s and this MS/MS scan event was repeated for the top 20 peaks in the MS survey scan. Singly charged ions were excluded from the MS/MS analysis and XCalibur software version 2.0.7 (Thermo Fisher Scientific, UK) was used for data acquisition.

### 2.7 Protein Identification and Quantification

The XCalibur raw files that contained the MS peptide sequencing information from the three parallel fractionations of each SILAC labelled protein fraction (total, nuclear, and cytoplasmic) were uploaded into MaxQuant software package (version 1.5.2.8). A list of tables returned by MaxQuant contained information representing the number of reconstructed proteins from the identified peptides (unique and razor), statistics on the peak detection, and normalized sample ratios (H/L).

For the integrated Andromeda search engine, protein sequences digested in silico (human FASTA files) (ftp://ftp.uniprot.org/pub/databases/uniprot/current_release/knowledgebase/proteomes/downloaded) were used. The following parameters were selected: two‐state SILAC included multiplicity of 2 for labeling with Lys0/Arg0 as light and Lys6/Arg10 as heavy proteins, a maximum number of three labeled amino acids per peptide, maximum of two tolerated tryptic missed cleavages, and carbamidomethyl Cys as a fixed modification. Identification parameters included decoy mode sequences, a value of 0.01 as the false discovery rate (1% FDR) at the peptide and protein level, minimal peptide length of six amino acids, a value of 1 was selected as minimal for identification (total peptides and razor or unique peptides), and a minimal Andromeda score of 0 and 40 for accepting an MS/MS spectrum of unmodified and modified peptides, respectively, in addition to imposing the desired FDR to consider the protein group identification in the final table. For relative normalized protein quantitation, the selected parameters were a minimal ratio count of 2, unique and razor peptides to calculate the protein ratios, and only peptides containing the specific modifications *N*‐terminal acetyl or oxidation of Met in addition to unmodified peptides.

The peptides and proteins tables were uploaded into Perseus (version 1.5.1.6) to rearrange the expression, numerical, and categorical columns, followed by merging the protein and peptide tables. The normalized ratio columns were log transformed prior to capture the quantity significance, called significance B (*p* < 0.05). Perseus was used to add annotation columns from specifically formatted files contained in the configuration folders.

### 2.8 Data Correlations and Merging Replica Datasets

The number of output datasets that were used in this study containing the identified and quantified proteins from the peptide sequencing MS raw files was obtained by MaxQuant. Three individual technical replicates for each sample type cytoplasm (C1, C2, C3), nucleus (N1, N2, N3), and total lysate (T1, T2, T3) were processed before merging the replicates in the denoted UNION dataset. The three technical replicates of each fraction were merged and processed in MaxQuant to correlate the distinct protein sequence groups and identify the total number for the union of consensus protein groups across the union dataset for the three replicates of each fraction (C‐UNION, N‐UNION, T‐UNION).

### 2.9 Selection of the Most Significantly Changed Proteins

From the merged dataset of the three sample types (C, N, T) of the 2418 identified proteins, two datasets of the most significantly changed proteins were obtained:

1. Overall “relaxed” response set (TAM‐270) was selected for those less abundant proteins identified with at least one peptide, ≥2 SILAC counts in the C, N, or T UNION, at least one SILAC ratio (S_n_, S_c_, or S_t_) with |log_2_(S)| > 1 (Table S1, Supporting Information).
2. Reliable response significance set (TAM‐108) for which all proteins in the 270‐TAM dataset contains only proteins identified by at least two peptides (one unique) and a minimum three SILAC ratio counts for the reliable quantification of the identified proteins (Table S2, Supporting Information). Based on adjusted *p*‐value below 0.05 (Sig B) and log2‐fold changes above 1 or below −1, both upregulated and downregulated proteins were obtained and their distribution of significance in the three sample types (C, N, T) is shown in Table S2, Supporting Information and used for the downstream bioinformatics/validation.

For the visualization and assignment of statistically represented GO‐enrichment function and enriched KEGG pathways Cytoscape (version 3.7.1) was used.^18^ The significantly enriched pathways were analyzed using Advaita Bioinformatic iPathwayGuide software (www.advaitabio.com/ipathwayguide.html) in the context of pathways obtained from the Kyoto Encyclopedia of Genes and Genomes (KEGG) database (Release 73.0+/03‐16, Mar 15)^19^ and For Gene (GO) annotation enrichment analysis, biological process (BP) and molecular function (MF) terms were obtained from the Gene Ontology Consortium database (2014‐Sep19)^20^ applying the classical overrepresentation approach to compute the statistical significance followed by corrected *p*‐value using Elim pruning method^21^ to remove the genes mapped to a significant GO term from more general (higher level) GO terms.

The redistribution of proteins shuttling between the subcellular compartments was carried out by 3D razor model described in detail in the previous paper.^14^

### 2.10 Confocal Fluorescence Imaging of Fixed Cells

To observe the changes in the cellular morphology in the treated and non‐treated cells, cellular (nuclear membrane(s), mitochondria, fibers, and nucleoli) and nuclear staining was combined using the Chromeo Red Fluorescent Fixed Cell Staining Kit (Active Motif Europe). Cells were grown to the desired confluence in DMEM‐F12 medium on coverslips (1.5 thickness, 13 mm diameter) inside a 24‐well plate. The phenol red containing medium was aspirated off and replaced with a fresh phenol free DMEM/F12 supplemented with 10% charcoal‐treated FBS for 48 h at 37 °C in a humidified incubator of 5% CO_2_; culture medium was replaced every 24 h. The control cells were treated with ethanol (vehicle) and the treated cells with 1 µm 4‐OHT and incubated further for 24 h at 37 °C per 5% CO_2_. Both control and treated cells were washed with cold PBS and fixed by adding 100% ethanol and the 24‐well plate was placed at −20 °C for 20 min (manufacturer's instructions). The fixed cells were washed twice with PBS and incubated with 1 µm of diluted cell stain solution in PBS at room temperature (protected from light) for 30 min. The stained cells on the coverslips were mounted on glass slides using MAXflour DAPI Mounting Medium and the coverslips were sealed with nail polish prior to imaging. The images were obtained using a Confocal Laser Scanning Module LSM 510 with ×63 oil immersion objective and the DAPI stained nuclei were detected using a standard DAPI filter set (370–410/435–485 nm). To detect the cellular stain in combination with stained nuclei, the fluorescent spectra were separated using a filter set (550–580/590–650 nm).

### 2.11 Immunofluorescence

MCF‐7 cells were seeded on coverslips (#1.5 thickness, 13 mm diameter) inside 24‐well plate grown under the same growth condition described in the florescence staining section. The cells were exposed to a dose of 1 µm 4‐OHT (stimulated cells) and vehicle treated (non‐stimulated control) for 24 h prior to fixation and immunolabeling. At room temperature, the cells were fixed on coverslips in 4% w/v paraformaldehyde for 10 min, permeabilized with 0.3% Triton X‐100 detergent for 5 min at room temperature, and blocked with 5% BSA in PBS for 1 h. The stimulated and non‐stimulated cells were incubated with anti‐MTCO2 antibody (ab79393, Abcam, Cambridge, UK, 5 µg mL^−1^) and anti‐CASPASE antibody (ab174847, Abcam, Cambridge, UK 1/250) overnight at 4 °C. The next day, a further 1 h incubation at room temperature was conducted with goat polyclonal secondary antibody to rabbit IgG—H&L (Green DyLight 488), pre‐adsorbed (ab96899, Abcam, Cambridge, UK) at 1/1000 dilution for primary anti‐MTCO2 antibody and 1/250 for primary anti‐CASPASE antibody. The unspecific binding was assessed by incubating the cells with only secondary antibody at 1/1000 and 1/250 dilutions for 1 h at room temperature. DAPI (D3571, Invitrogen) (excitation/emission (nm) 358/461) was used at 1.43 µm concentration to stain the nuclei of fixed cells for 5 min in dark at room temperature. Prior to imaging with Confocal Laser Scanning Module LSM 510 (×63 oil immersion objective), coverslips were mounted on glass slides using Vectashield antifade mounting medium (Vector Labs, H‐1000) and sealed with nail polish.

## 3 Results

### 3.1 Exposure of MCF‐7 Cells to Tam

We prepared MCF‐7 cells according to previously published procedures.^14^, ^17^, ^22^ Their response to Tam was measured for total change in protein abundance and for compartmental changes in protein abundance in the nuclear and “cytoplasmic” (nucleus‐depleted) compartments.^12^, ^13^, ^23^ The purity of the nuclear/cytoplasmic fractions obtained with the subcellular fractionation protocol previously optimized^13^, ^14^, ^16^, ^17^, ^22^ were routinely tested using western blotting.^14^, ^17^, ^22^ Stringent purification of organelles (e.g., nucleus) was not attempted since stringent organelle isolation procedures can lead to loss of ability to monitor important characteristics of dynamic cellular function. We preserved the ability to study dynamic features by using differential isotope labeling of cells with/without exposure to Tam and subcellular protocols during data analysis.^16^, ^24^

A concentration of 4‐OHT (1 µm) was previously characterized as a cytostatic dose for various cell lines and used in different laboratories. It was originally selected for complete inhibition of estrogen dependent ZR‐75‐1 cells without influencing the growth of Tam‐resistant derivatives.^25^ This dose has been routinely used because of the absence of complete toxicity, to effectively counter variations in estrogen content of different batches of bovine serum,^26^ and because an IC50 concentration of a drug that is required for 50% inhibition of in vitro Tam is 1 µm.^27^

Exposure of cells to 1 µm 4‐OHT for 24 h was used in the present experiments. The dose dependent (0.0–2.0 µm) impact of 4‐OHT treatment on the survival rate of MCF‐7 cells showed that the chosen dose (1 µm) was of a cytostatic effect (non‐lethal) to MCF‐7 cells, whereas a survival rate of less than 50% of the seeded cells was associated with higher doses of 1.5 and 2.0 µm 4‐OHT.

Although a restoration of the ERα complex probably linked to the half‐life of OHT was recently observed at the latest time point of 24 h by interactome dynamics studies,^28^ a longer stimulation up to 36 h was adopted by others for the investigation of late‐response proteins,^29^ in agreement with results on late‐response genes at 24 h by transcriptomic approaches.^30^

We initially published the proteomics study of subcellular protein distribution^22^ in MCF‐7 cells followed by subcellular proteomics under the E2 exposure,^14^, ^15^ OHT exposure, and OHT+E2 exposure (manuscript in preparation). All experiments using MCF‐7 were done in the same manner and the tests were performed accordingly with the details given in previous papers.^14^, ^15^, ^22^

**Figure 1:**
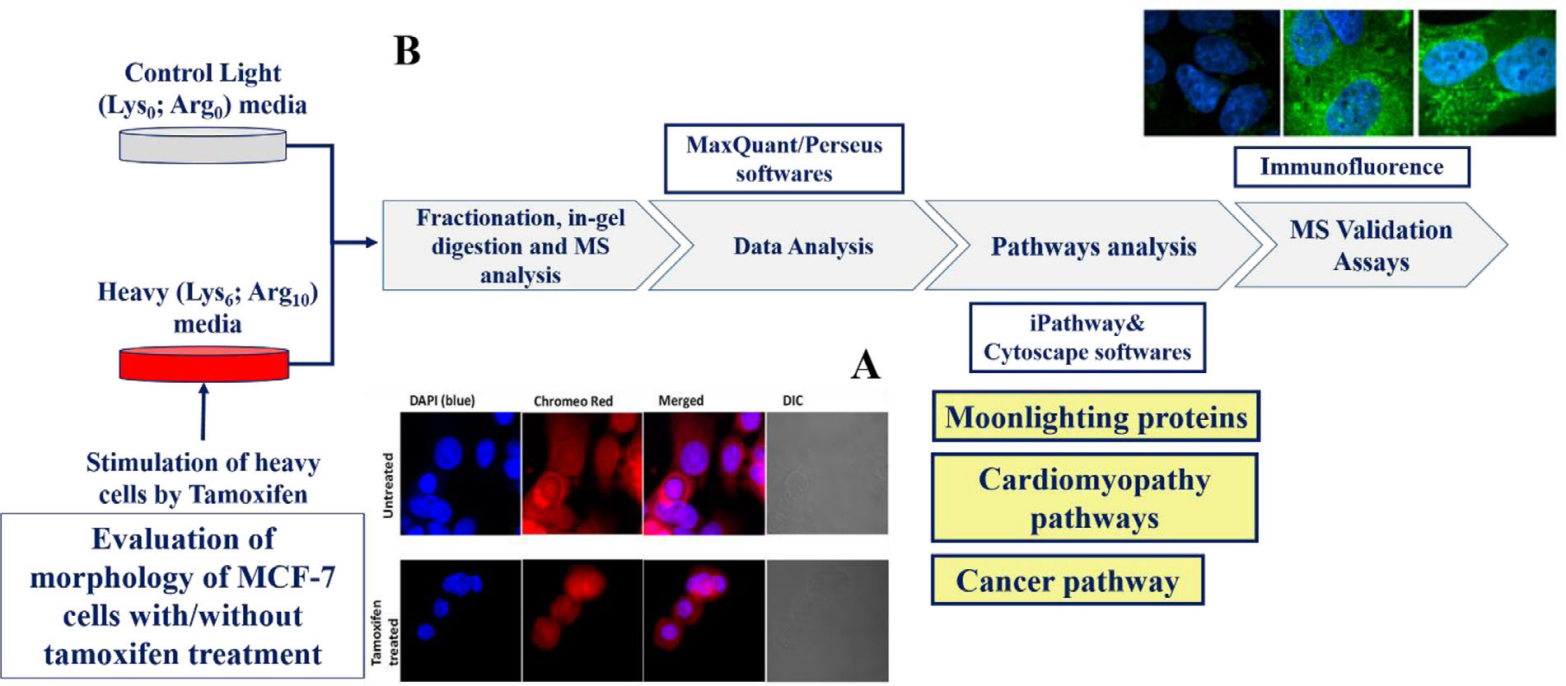
Immunofluorescent assay test of morphological changes of MCF cells stimulated with tamoxifen. B) Schematic summary of the experimental workflow.

As a routine control for the present preparations, we checked the morphology of stimulated/unstimulated cells using fluorescence staining with a proprietary dye that can label nuclear membrane(s), mitochondria, fibers, and nuceleoli in fixed cells. Confocal imaging revealed no remarkable changes in overall cell morphology in the 4‐OHT–treated MCF‐7 cells compared to untreated cells (**Figure** 1A).

### 3.2 Changes in Abundance for the Total, Nuclear, and Cytoplasmic Fractions

The experimental strategy by SILAC labeling, stimulation, MS quantification, and the software used for pathway analysis are reported in Figure 1B.

Experimental measurements of protein abundance ratios (SILAC ratios) between stimulated/unstimulated cells were obtained for three samples: 1) an unfractionated, total cell lysate (T), 2) a nucleus‐enriched sample obtained by subcellular fractionation (N), and 3) the corresponding nucleus‐depleted sample (C), which we refer to as the cytoplasm in the following text. The corresponding SILAC ratios provide measures for each protein of the overall change in total cellular abundance (S_t_), or of the localized change in abundance in the nuclear (S_n_) or cytoplasmic (S_c_) subcellular compartments (Table S3, Supporting Information).

For each sample type (C, N, T), we measured three replicates. Joint processing of all nine datasets identified 4276 different proteins. Using conservative criteria that a given sample type reliable quantification requires: a) two identified peptides and b) at least five SILAC ratio counts for the union over the three replicates, 2418 proteins were accepted for the analyses described below. The overall changes in abundance for these proteins are shown in Figure S1, Supporting Information. The inset tables show the number and percentage of proteins with >fourfold or >twofold changes in total abundance (S_t_), nuclear compartment abundance (S_n_), and cytoplasmic compartment abundance (S_c_). The tables also show for each distribution the total number of proteins included, the median number of SILAC ratio counts, and the number of proteins with smaller numbers of SILAC ratio counts. The striking features of the distributions are: a) only 57 proteins (≈2% of proteins) show twofold increase/decrease in total abundance, b) for the nuclear compartment, 576 proteins (≈31% of nuclear proteins) show >twofold decrease in nuclear abundance while only 4 proteins (≈0.2%) show >twofold increase in nuclear abundance, c) for the cytoplasmic compartment, 98 proteins (≈7% of cytoplasmic proteins) show >twofold decrease in cytoplasmic abundance while 297 proteins (≈23%) show >twofold increase in abundance. Comparison with a smaller set of 2551 more abundant proteins with at least two identified peptides and three ratio counts in each replicate (Figure S1, Supporting Information) verifies that the distributions are not distorted by the inclusion of less abundant proteins. We concluded that the dominant feature in the response of MCF‐7 cells to Tam is *not* changes in the total abundance of proteins by transcription/translation/degradation, but instead large numbers of proteins with substantial changes in their spatial distribution over the cytoplasmic and nuclear compartments.

### 3.3 Metanalysis of the Functional Pathways

The 270‐TAM set (see Section 2.9) included less abundant proteins identified with at least one peptide, ≥two SILAC counts in the C, N, or T UNION, at least one SILAC ratio (S_n_, S_c_, or S_t_) with |log_2_(S)| > 1 was used to search for additional proteins potentially associated with specific pathways. These protein lists were mapped to the approved gene symbols and reloaded into the iPathway Guide software. The analyses were carried out for the union of the DA proteins (270‐TAM) as well as for the individual sets of C, N, and T proteins.

Overall there were 67 significantly impacted pathways (270‐TAM) with 19, 43, and 28 pathways for the C, N, and T proteins subsets, respectively (Table S4, Supporting Information). The *p*‐values were corrected for multiple comparison using a FDR < 0.05 for the number of significantly impacted pathways in each sample type. The top ranked pathways, in each sample type, were selected after eliminating false positives (5% FDR) (**Table** 1). This analysis showed that protein changes in abundance with 4‐OHT across multiple conditions were involved in pathways related to metabolism, signal transduction, growth and proliferation, and development.

**Table 1:** Top KEGG pathways significantly impacted in the three sample types CNT (TAM‐270 dataset) and their associated corrected (FDR) *p*‐values

Pathway name	Pathway Id	*p*‐value	*p*‐value (FDR)
Total lysate fraction			
Metabolic pathways^a^	1100	9.48E−07	9.13E−05
Oxidative phosphorylation^a^	190	2.50E−06	9.13E−05
Non‐alcoholic fatty liver disease (NAFLD)	4932	6.60E−06	1.78E−04
ECM‐receptor interaction	4512	6.37E−05	0.001
Fructose and mannose metabolism^a^	51	3.55E−04	0.005
Cytoplasmic Fraction			
Hypertrophic cardiomyopathy (HCM)^a^	5410	7.67E−05	0.008
Dilated cardiomyopathy	5414	3.16E−04	0.016
Cardiac muscle contraction^a^	4260	0.001	0.036
Calcium signaling pathway	4020	0.001	0.036
Adrenergic signaling in cardiomyocytes	4261	0.003	0.052
Nuclear Fraction			
Circadian entrainment	4713	6.20E−05	0.007
Dilated cardiomyopathy	5414	5.59E−04	0.016
Adrenergic signaling in cardiomyocytes	4261	0.001	0.018
Hypertrophic cardiomyopathy (HCM)^a^	5410	0.002	0.02
Pathways in cancer	5200	0.004	0.039

Full list is Table S4, Supporting Information

a The *p*‐value corresponding to the pathway was computed using only overrepresentation analysis.

In the total lysate experiment (T), five top ranked pathways were identifed as signifcantly impacted and indicate that the significantly changed proteins constitute overrepresentation of metabolic pathways and oxidative phosophorylation (*p* = 9.1 e^−05^), as well as fructose and mannose metabolism (*p* = 0.005). Proteins that were upregulated/downregulated belonged to the total lysate fraction (T), where seven proteins, ALDOA (C), IDH1 (C), LDHA (N), MTCO2 (C, N), TKT (N, T), EBP (C), NANS (C, T), were upregulated in the C and/or N fractions with or without change in total aboundance. Seven proteins, CKMT2 (C), NDUFV3 (N), OAT (N), P4HA1 (C), TST (C, N), NNT (N), and PGLS (C) were dowregulated in the subcellular fractions (S_n_ and/or S_c_) without change in the total aboundance.

Based on the statistical selection criteria (significance B < 0.05), glutathione synthetase (GSS) were quantified in both C, T samples with significant upregulation in the T sample (S_t_ = 5.244 and *p* = 0.002), only. Hence, this protein was not quantified in the nuclear fraction; it was filtered out of the stringent list (108‐TAM) showing the distribution of significance in the three sample types based on the selection criteria described in the material and method section. GSS has among other functions a protective role in the cells exposed to the oxidative stress by preventing the oxidative damage. We note that MTCO2 has shown significant increase in the C and N sample and NDUFV3 in N without change in the total abundance associated with either of the two proteins.

Pathways that are related to signal transductions, growth, and development show predominant changes in the subcellular compartmental redistribution (C and N) rather than in total abundance changes.

### 3.4 Significant Changes of the Proteome after Exposure to 4‐OHT

**Figure 2:**
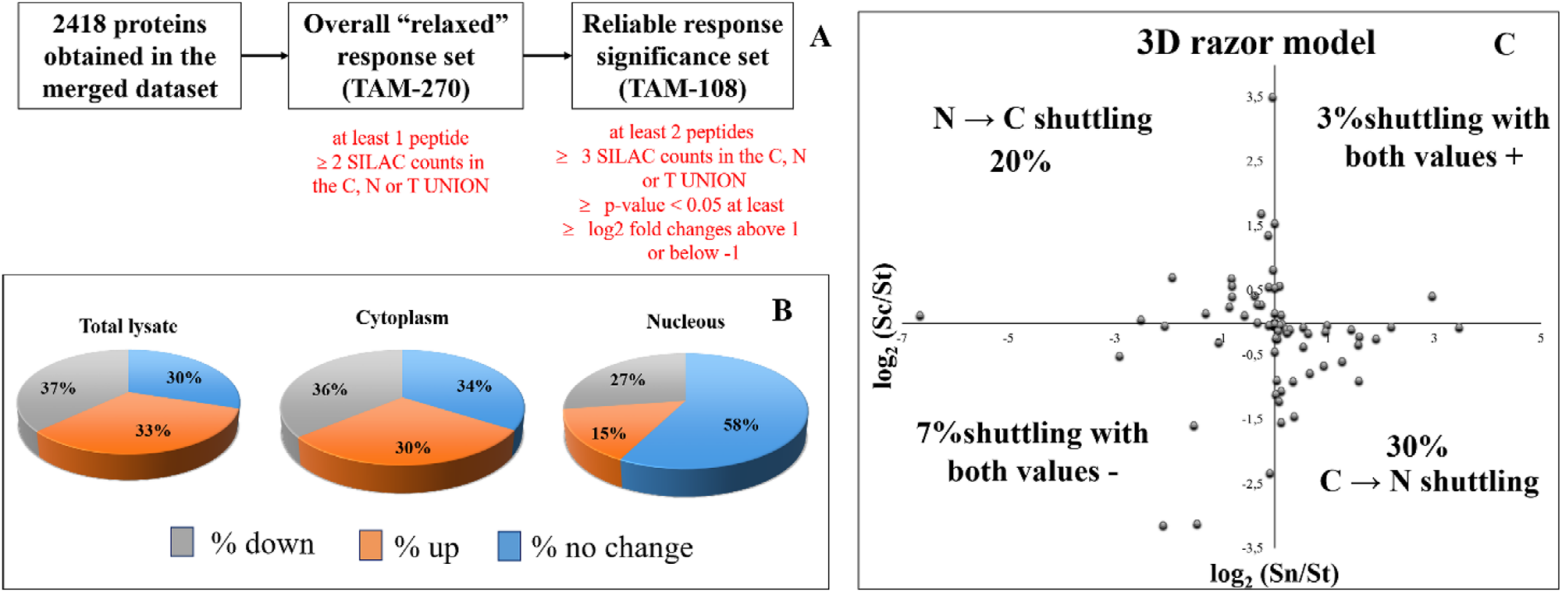
Filtering steps of 2418 proteins obtained in the merged dataset and the selection of the most significant 108 proteins for at least one of the three samples. B) Percentage of up/down or no change of significant proteins described by pie charts for total lysate, cytoplasm and nuclear compartment within the 108 datasets. C) Razor model within the orthogonal 3D space {Sn/St, Sc/St, St}, the theoretical distribution plane {Sn/St, Sc/St} for different values of ƒ_u_ (the fraction of protein in the nucleus in the unstimulated cells) as the fraction of the protein in the nucleus in the stimulated cells (fs) varies over 0 < ƒ_s_ < 1. Conservation of mass restricts the cellular response to two quadrants corresponding to N → C or C→ N redistribution of the protein upon stimulation.

Within 270‐proteins there was a dataset that corresponds to the core response (108‐TAM with most significant changes in response to Tam treatment (Table S2, Supporting Information) following the filtering process reported in **Figure** 2A. The 108‐TAM dataset corresponds to highly reliable proteins that are: a) identified with ≥2 MS‐sequenced peptides, b) quantified with ≥3 SILAC counts in at least two replicates, and c) have at least one *p*‐value < 0.05 in one of compartment subcellular (S_n_, S_c_, or S_t_) SILAC ratio following. The percentage of up/down or no changes of 108 significant proteins is represented in pie charts for each for total lysate, cytoplasm, and nuclear compartment (Figure 2B).

**Figure 3:**
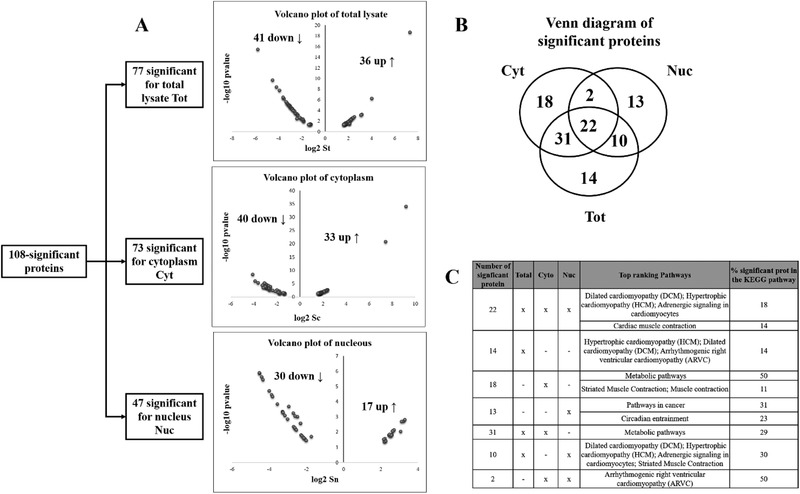
Volcano plot of the most significant proteins described by *p*‐value and normalized ratio H/L registered in total lysate and nuclear/cytoplasm compartments, within 108 datasets. B) Venn diagram of the distribution of significant proteins in cytoplasmic, nuclear, and total lysates and the number of those shared between the different cellular samples. C) Distribution of KEGG pathways where the proteins of each sample or those shared between the different samples are involved.

It is notable that of the 108 proteins, 70% corresponds to significant changes in S_t_. As a consequence of basal distribution between compartments, 30% proteins show significant changes in compartmental abundance (S_n_ or S_c_) without significant changes in S_t_ (Figure 2B). Almost 50% of the 77 proteins changing in total sample is down‐ or upregulated as shown from the Volcano representation of normalized ratios H/L plotted with the relative *p*‐values (**Figure** 3A). Moreover, 14 are exclusively belonging to the total fraction, while 31, 10, and 22 are shared only with cytoplasm, only with nucleus or with both of them, respectively, as reported in Venn diagram (Figure 3B). A similar distribution is observed in cytoplasm while only 40% of proteins show changes in the nuclear compartment (Figure 2B). Among the dataset of 108 proteins, roughly 30% of them are exclusively changed in the nuclear compartment (Figure 3B). For example, response to 4‐OHT strongly reduces the abundance of ANXA1 in the nucleus (S_n_) with only very moderate changes in total or cytoplasmic abundance (Table S1, Supporting Information). Same speculation can be made for the guanine nucleotide‐binding protein subunits, GNA13 (S_n_ = 3.1), GNAQ (S_n_ = 2.6), GNAS (S_n_ = 2.5), GNB1 (S_n_ = 2.6), which appeared to be overrepresented up to ninefold compared to non‐stimulated sample in the Circadian entrainment pathway and in cancer pathway (Figure 3C) (see Section 4). Further, interesting results derive from the application of the razor model within the orthogonal 3D space {Sn/St, Sc/St, St} described by the theoretical distribution 2D plane {Sn/St, Sc/St} (Figure 2C). Roughly 20% proteins are translocated from N → C, as previously observed in MCF‐7 cells stimulated with E2.^14^ In contrast to the translocation of protein in response to E2, a higher percentage of proteins moved in opposite direction (C→ N) as a result of TAM stimulation (Figure 2C). Conservation of mass restricts the cellular response to two quadrants corresponding to N → C or C→ N (Figure 2C).

Quantitative data show that, after the Tam stimulation, a marked decrease of compartmental abundance were recorded for CASP14 with a more than fourfold decrease in the nuclear compartments (S_n_ −2.488107, *p*‐value 0.000767178) and a more than twofold decrease in the cytoplasmic one (S_c_ −1.787169, *p*‐value 0.008297533). In the absence of changes in total abundance, CASP14 was significantly redistributed in cytoplasm (N → C). In the case of MTCO2 protein, a greater than fourfold increase of nuclear abundance (S_n_ 2.704, *p*‐value 0.0051) and the decrease in cytoplasmic one (S_c_ 1.819, *p*‐value 0.0036) pointed out its opposite redistribution C → N. These relevant proteins, CASP14 and MTCO2, were also chosen for MS validation by confocal microscopic analysis (Figure S2, Supporting Information).

The subcellular translocation of DES appears to be more balanced and strong N → C redistribution results in significant decrease/increase in N/C compartmental abundance with little change in total abundance. Overall, the observation of many proteins with strong changes in S_n_ or S_c_ without significant change in S_t_ indicates that spatial redistribution of key proteins is an important component of response to 4‐OHT.

### 3.5 Cardiac and Cancer Pathway in Response to Tam

Among the top ranked pathways, different distributions of seven pathways were observed for the cytoplasm (50%), nucleus (less than 10%), and total lysate (more than 40%) (**Table** 2).

**Table 2:** Enrichment KEGG pathway for reported proteins with an FDR < 0.05

Matching proteins	Pathway ID	Pathway description	False discovery rate	Tot Up [%]	Tot Down [%]	Cyto Up [%]	Cyto Down [%]	Nuc Up [%]	Nuc Down [%]
DES,MYH7,MYL2,MYL3,TNNT2,TTN	5410	Hypertrophic cardiomyopathy	3,99E−09	16,7	83,3	—	83,3		100
DES,MYH7,MYL2,MYL3,TNNT2,TTN	5414	Dilated cardiomyopathy	3,99E−09	16,7	83,3	—	83,3		100
MTCO2,MYH7,MYL2,MYL3,TNNT2	4260	Cardiac muscle contraction	2,06E−06	20	80	20	60	20	80
CALML5,MYH7,MYL2,MYL3,TNNT2	4261	Adrenergic signaling in cardiomyocytes	6,69E−05	—	100	—	80		100
ACTN2,DES, DSP	5412	Arrhythmogenic right ventricular cardiomyopathy	0,0386	—	100	—	100	—	100
GNAS, GNA13, GNB1	4713	Circadian entrainment	7,00E−03	100	—	100	—	100	—
GNAS, GNA13, GNAQ, GNB1	5200	Pathways in cancer	0039	100	—	75	—	100	—

**Figure 4:**
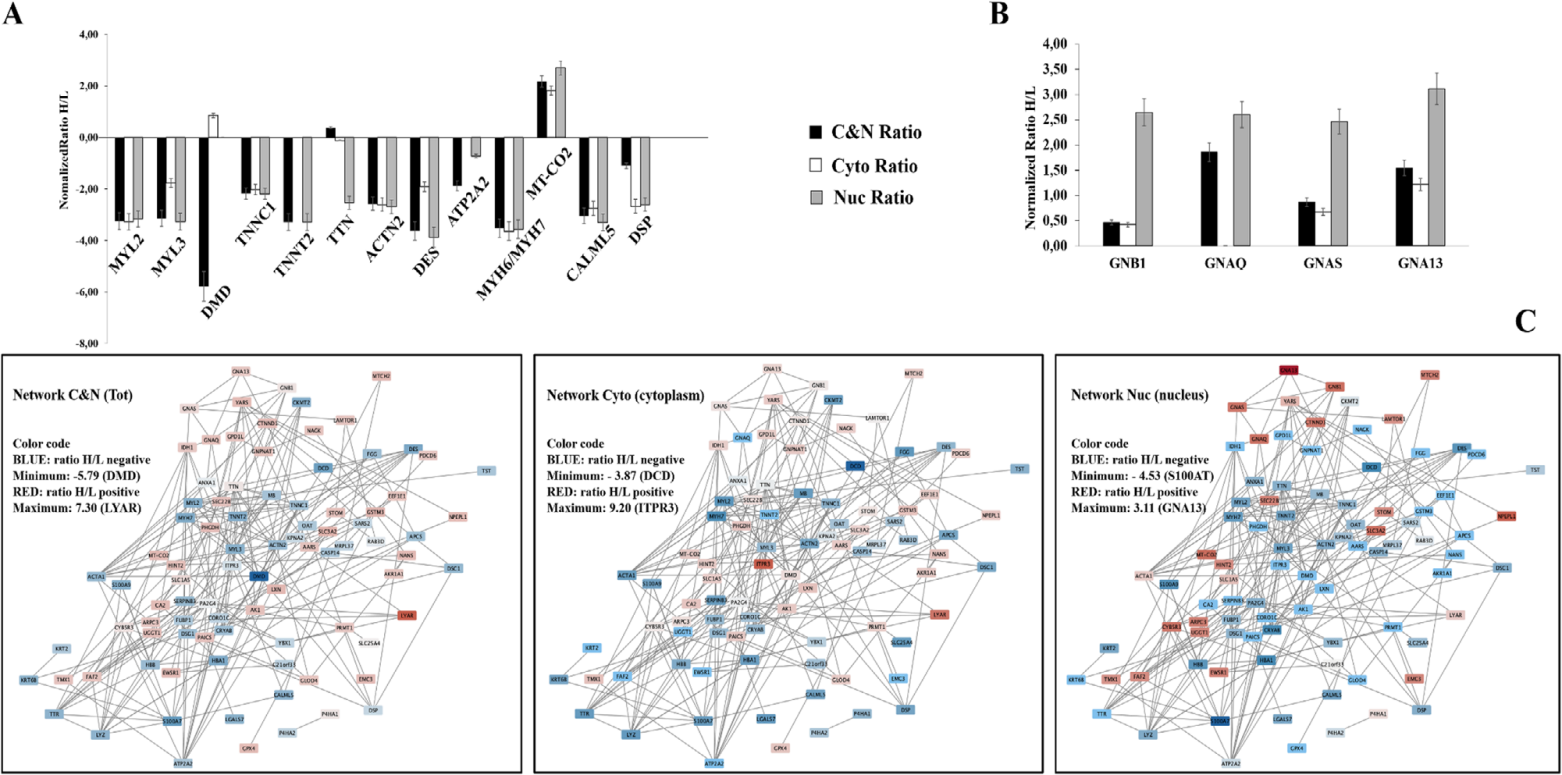
Distribution of protein expression of total lysate (blue bar), cytoplasmic (orange bar), and nuclear compartments (gray bar) in A) cardiomyopathy enrichment KEGG pathways, B) and Circadian entrainment or cancer enrichment KEGG pathways, and C) Cytoscape analysis for total lysate, cytoplasmic, and nuclear samples (C).

Among 108 proteins analyzed by the Cytoscape network (**Figure** 4C), 17 were involved in cardiomyopathy enrichment KEGG pathways (Table 2). Fourteen of those shared hypertrophic, dilated, and/or arrhythmogenic right ventricular cardiomyopathy pathways or they were proteins involved in cardiac muscle contraction and adrenergic signaling in cardiomyocytes (Table 2; Figure 4A). Most of these proteins were downregulated in all the subcellular datasets (C and N), including the total lysate as shown in Figure 4A. We notice the exception of DMD that was upregulated in cytoplasmic fraction without showing changes in total lysate while TTN showed to be upregulated only in the total lysate. The MTCO2, known for its crucial role in the oxidative phosphorylation and other pathways displays an upregulation for all datasets indicating its moonlighting function.

Other proteins encoded by MYBPC3 and TNNI3 genes were particularly downregulated in the nuclear compartment (*p*‐value = 0.005 for MYBPC3 and 0.008 for TNNI3) showing fourfold lower abundance than in the unstimulated samples (Table S3, Supporting Information).

Circadian entailment pathway (Table 2) represented by the guanine nucleotide‐binding protein (GNAQ, GNAS, GNB1, GNB2) was upregulated in all datasets, with an exception of GNAQ protein showing no change in cytoplasm compartment (Figure 4B). These proteins are also reported to be upregulated in cancer pathway (Table 2) indicating possible regulatory role in breast cancer cell invasion by G‐protein coupled receptors (GPCRs) signaling.^31^

## 4 Discussion

Response of MCF‐7 cells to Tam is known to induce an adaptive response in extensive changes in abundance for hundreds of proteins^32^ and phosphoproteins.^33^ However, the studies have shown that the extensive spatiotemporal redistribution of subcellular proteins is an increasingly important evidence of cellular response to various stimuli.^23^ The present experiments reveal dynamic redistribution of numerous proteins in the context of Tam stimulation and suggest that many aspects of cellular response to Tam are still incompletely understood.

From the large MaxQuant dataset, we selected an overall response list of proteins (270‐TAM) and a more reliable response (108‐TAM) for which more significant changes actually occurred in response to the Tam treatment (see Section 2). Within the108 proteins, two major protein groups with different response to 4‐OHT were observed: a) Proteins with exclusive changes in total abundance without redistribution in the nucleus/cytoplasm. They represent 20% and give a low contribution to the cellular response; b) Proteins with abundance changes only in one of two compartments (nucleus or cytoplasm); c) Proteins with significant abundance changes in both compartments (nucleus/cytoplasm) with no change in total abundance (20%). Although the (a) group is equally important for the large‐scale studies, we are more interested in (b) and (c) groups, representing the majority (70%) of proteins responsive to 4‐OHT.

We observed in group (b) MTCO2 known for its distinct mitochondrial role in energy‐generating process and oxidative phosphorylation. It binds directly to cytochrome c and is known for its role in apoptosis regulation.^34^ It has been suggested that a small fraction of cytochrome c, more weakly attached to inner membrane of mitochondria, is presumably more available for compartmental shuttling within cell. Its redistribution from mitochondria to cytosol occurs in response to pro‐apoptotic stimuli through the interaction with Apaf‐1 leading to caspase cascade events.^31^ In addition, cytochrome accumulation in nucleus compartment triggers massive translocation of acetylated histone H2A from nucleus to cytoplasm with irreversible consequence on chromatin condensation.^35^ The cytoplasm cytochrome can further block calcium dependent inhibition of IP3 receptor on the endoplasmic reticulum and increase the calcium release from ER that in turn activates cytochrome c release.^36^ As the subcellular proteomics studies of cellular dynamics revealed the frequent existence of numerous “moonlighting” proteins that escape the classic concept of one protein → one compartment → one function^24^ the depletion or altered expression of some of OXPHOS proteins (MTCO2, COX I, III) that has also been implicated in tumorigenic transformation^37^ appears to indicate their “moonlighting” functioning.

Another moonlighter, downregulated in our cytoplasm and nucleus fractions, is CRYAB, a potential biomarker of several tumor included in triple‐negative breast cancer (TNBC).^38^ CRYAB is a major structural protein in ocular lens for transparency maintenance in nonlenticular tissues functioning as cytoprotective molecular chaperone capable of preventing non‐specific protein interactions under particular cellular stress. It specifically binds and corrects intracellular misfolded/unfolded proteins such as vascular endothelial growth factor promoting angiogenesis in metastatic event.^39^ An anti‐apoptotic function by inhibiting the autoproteolytic cleavage of caspase3,^40^ or by directly interacting with the pro‐apoptotic Bax and Bcl‐xs^41^ in response to diverse stimuli has also been attributed to this protein. Moreover, in response to heat stress, CRYAB translocates from cytoplasm to nucleus and complexed to F‐actin participates in regulation of cytoskeletal stability and dynamics.^42^ The shuttling of CRYAB to mitochondria is also known to inhibit cytochrome c release into cytosol during MI by binding to VDAC1 channel.^43^

We note discrepancies between the GO annotation and the present subcellular locations confirming the underestimation of subcellular annotations related to the GO CC database. The high dispersion of proteins over multiple locations as recently reported in large‐scale proteomics papers^14^, ^17^, ^22^ suggests that the real spatial distribution of cellular functions is much more complicated than it is annotated in the current GO CC. In the present paper, for more than 60% of proteins (all 108 dataset), there is at least one location omitted in GO annotation. While for some proteins the additional subcellular location found is not well documented but for others it is.

Within the (c) group, an additional nuclear location to the GO annotation in cytoplasm and/or in extracellular region has been found for MTCO2, DES, HBB, DCD (**Table** 3). For proteins annotated with a single location such as MYL3 (Cytosol), FUBP1 (Nucleus), LYAR (Nucleus) CALML5 (extracellular vesicular exosome) a subcellular redistribution between nucleus and cytoplasm have also been found following the 4‐OHTam treatment (Table S2, Supporting Information). For example, LYAR, a zinc finger protein, more frequently found in the nucleolus for promoting cell growth by preventing nucleolin self‐cleavage,^44^ has also been associated to cytoplasmic ribosomes in human cancer cells.^42^ From the cytoplasmic compartment, Lyar protein dissociates from the cytoplasmic 60S ribosomial subunit shuttling rapidly to the nucleolus for absolving nuclear functions. The abnormal expression of these ribosome‐interacting proteins has been associated to the deregulation in growth and viability of malignant neoplasms.^45^ Studies entirely referring to GO‐database inevitably suffer from the limitation and incomplete information on protein functions and their locations. In this context, the finding of new locations for subcellular proteins should be a lead for further studies.

**Table 3:** List of 19 proteins showing significant changes in compartmentalized abundance (S_c_, S_n_) in the absence of significant changes in total fraction

Gene symbol	GO‐Cellular component	S_c_ union	S_n_ union	log(S_n_/S_c_)	Distribution
SLC3A2	Nucleus, cytoplasm, plasma membrane, membrane, extracellular vesicular exosome	1.396	2.637	1.241	C→N
MTCO2^a^	Mitochondrion, mitochondrial inner membrane, membrane, extracellular vesicular exosome	1.819	2.704	0.884	C→N
MYL3	Cytosol	−1.768	−3.263	−1.496	N→C
SLC25A4	Nucleus, mitochondrion, mitochondrial inner, and plasma membrane	−3.033	−2.169	0.864	C→N
MYH6	Nucleus, cytoplasm, cytosol	−3.638	−3.568	0.070	C→N
DSP	Nucleus, mitochondrion, cytoskeleton intermediate filament plasma membrane, cell−cell junction extracellular vesicular exosome	−2.667	−2.604	0.063	C→N
DES	Cytoplasm, cytosol, cytoskeleton	−1.911	−3.879	−1.968	N→C
CASP14^a^	Nucleus, cytoplasm, extracellular vesicular exosome	−1.787	−2.488	−0.701	N→C
HBB	Extracellular region, cytosol, extracellular vesicular exosome	−2.522	−2.812	−0.291	N→C
HBA2	Extracellular region, cytosol, extracellular vesicular exosome	−2.824	−4.035	−1.211	N→C
DCD	Extracellular region, extracellular vesicular exosome	−3.879	−4.513	−0.634	N→C
DSG1	Cytosol, plasma membrane, membrane, cell−cell junction, extracellular vesicular exosome	−2.571	−2.212	0.359	C→N
DSC1	Plasma membrane, membrane, gap junction, extracellular vesicular exosome	−2.594	−2.606	−0.012	N→C
GNA13	Nucleus, plasma membrane, membrane, extracellular vesicular exosome	1.221	3.115	1.894	C→N
TST	Extracellular space, mitochondrion, mitochondrial inner membrane, mitochondrial matrix, extracellular vesicular exosome	−2.131	−1.456	0.675	C→N
FUBP1	Nucleus	−1.798	−2.080	−0.282	N→C
LYAR	Nucleus, nucleolus	7.707	7.096	−0.611	N→C
CALML5	Extracellular vesicular exosome	−2.752	−3.294	−0.543	N→C
MTCH2	Nucleus, mitochondrion, mitochondrial inner membrane, membrane, extracellular vesicular exosome	1.489	2.220	0.730	C→N

a Indicates the proteins chosen for MS validation by immunofluorescence.

### 4.1 Cardiomyopathy Pathways in Response to Tam

Cardiomyopathies are a heterogeneous group of diseases including hypertrophic cardiomyopathy, dilated cardiomyopathy, and arrhythmogenic cardiomyopathy characterized by a wide range of clinical manifestations, heart morphology, prognosis as well as varying genetics. Mutations in the *MYH6* and *MYH7* genes occurred in patients with both hypertrophic cardiomyopathy and dilated cardiomyopathy.^46^ In hypertrophic cardiomyopathy, roughly 70% of these mutations were related to genes encoding cardiac β‐myosin heavy chain (MYH7), cardiac myosin‐binding protein C (MYBPC3) or cardiac troponin (TNNT2, TNNI3), and actin (ACTC).^47^, ^48^ Moreover, the light chain of myosin plays a regulatory role in cardiac muscle contractions by binding Ca^2+^ ions at activating concentrations or binding actin as it occurs in the *N*‐terminal domain of Myl3 allowing it to contribute to force‐generating myosin cross‐bridges.^49^ These proteins resulted to be expressed differently in the atrial (Myl1) or ventricular tissue (Myl2, Myl3, and Myh7).^50^ Eleven significant pathways were enriched by 757 genes differentially expressed in specific regions of the heart with 475 genes in the atrial samples and 282 in the ventricular tissues.^50^ In this contest, the Tam produced a significant reduction of cardiac hypertrophy, bradycardia, and oxidative stress in hypertrophic rats subjected to the treatment.^51^ It appears that Tam have a crucial role in the inhibition of protein kinase C responsible for the hypertrophic gene regulation and in inducing less increase in cardiomyocyte diameter and lower reduction in extracellular space.^51^

In addition to this direct effect of Tam on cardiomyocyte, there is an evidence of its significant impact on the cellular lipid metabolism. The response to 4‐OHT treatment investigated on young cycling female rats showed a substantial impact on glycogen storage and on the reduction of cholesterol serum level inducing a slowdown in age dependent increase in body weight.^52^ A similar effect has been observed in healthy postmenopausal women where a reduction of serum lipoprotein, LDL, and cholesterol by 34%, 19%, and 12%, respectively, was observed as a consequence of 4‐OHT treatment, without changes in the HDL, its subfractions, triglyceride, and apolipoprotein A1 levels content.^53^ At cellular level the direct lipid dysregulation by 4‐OHT derived by binding to the microsomal antiestrogen is known to affect the cholesterol metabolism.^54^ The reduction of free cholesterol in serum indirectly induced by 4‐OHT can be correlated to the increase of LDL receptor expression inducing a block of the transport of cholesterol to the endoplasmic reticulum and the subsequent inhibition of the SREBP‐2 pathway by impairing the egress of LDL‐derived cholesterol from late endosomes/lysosomes.^55^

The protective role of 4‐OHT against cardiovascular diseases appears to be indirectly due to lipid reprogramming mediated by drug^56^ and to the cholesterol pathway association to altered gene expression patterns that can partially explain the Tam resistance of cells.^57^ Thus the downregulation of most proteins showed in Figure 4A could indicate a positive impact of Tam on cardiomyopathy pathways in MCF‐7 cell.

### 4.2 G‐Protein Coupled Receptors and Cancer Pathway in Response to Tam

Some proteins of the guanine nucleotide‐binding protein subunits GNAQ (Gqα), GNAS (Gs‐α), GNB1(Gβ1), and GNA13 (Gα) were overrepresented in the Circadian entrainment pathway as well as in cancer pathway. These findings imply the importance of the heterotrimeric G‐proteins in transducing the non‐genomic and genomic (transcriptional) signaling in response to the clinical relevant dose of (1 µm) 4‐OHT.^58^ In breast cancer cells, the Tam is known to act as agonist on GPCR 30 (GPR30)^59^, ^60^ and GPCR30/G‐protein coupled oestrogen receptor‐1(GPR30/GPER‐1).^59^, ^60^ GPCRs are cell surface receptors with wide spectrum of functions in physiological and pathophysiological processes that range from tumor formation to the spread of cancer cells. Ligand dependent GPCRs such as SDF‐1, thrombin, LPA, S1P, and endothelin receptors were found to have pivotal roles in many forms of tumors including prostate and breast cancer.^61^ Their downstream signaling cascades are transduced through G‐Alpha12 (GNA12) and G‐Alpha13 (GNA13) subunits constituting the subfamily of heterotrimeric G‐proteins. Indeed, invasion and metastasis have been attributed to GNA proteins in many cancer types.^62^ Some findings reported that GNA13 is highly upregulated in the aggressive form of breast cancer cells^31^ and restoration of GNA13 activity has been shown to enhance the progression and invasion characteristics of prostate and gastric cancer cells.^63^ We found that under 4‐OHT stimulation GNA13 were highly expressed in the cytoplasmic and nuclear fraction and in the total lysate while GNAQ were upregulated in the total and nuclear fraction without any change in cytoplasmic compartment (Figure 4B). In addition to the localization of seven transmembrane receptors to the plasma membrane, subcellular localization of GPR30 was reported to endoplasmic reticulum,^64^ and possibly in Golgi^65^ in addition to suggested shuttling of GRP30.^66^

The ligand binding to the oestrogen receptors (termed ERα and ERβ) initiates a sequence of events that result in genomic (transcriptional) and non‐genomic signaling processes. GPR30, found to be expressed in 50% of cancer patients could induce the non‐genomic mediated tumour progression through mitogen‐activated protein kinase (MAPK) even where ERs are absent or blocked.^67^, ^68^ The time frame for genomic and non‐genomic responses is not the same, the genomic one is slower than non‐genomic response and lasts hours to days while the later lasts seconds to minutes. The rapid (non‐genomic) estrogen response transfers the signals/information to the subsequent effectors in the cascade that includes second messengers (Ca2+, cAMP, and NO), and kinase activation (e.g., PI 3‐kinase, Akt, and MAPK).^59^ The discoveries of these two receptors has complicated the interpretation of their actions and functions. Their localizations in the cytosol and/or nucleus justifyes the slow response of genomic (transcriptional) actions. On the other hand, the non‐genomic actions (rapid) that involve signaling transactions were linked to the existence of membrane‐associated oestrogen receptors that are structurally distinguished from the classical ERs.^69^ A number of studies have demonstrated that Tam and its metabolites show profound effect on breast cancer proliferation through GPR30 implying the role of this protein in the acquired resistance to targeted Tam treatment in breast cancer patients.^70^, ^71^, ^72^, ^73^ One of the suggested mechanisms that induce the Tam resistance is the activation of the epidermal growth factor receptor (EGFR). The expression of GPR30 in 50% of ER +  breast cancer patients,^68^ together with the overexpression of EGFR in acquired resistance suggests that Gβγ subunit protein of GPR30 may induce the GPR30/EGFR signaling cascade.^74^ The downstream ligand‐activated GPR30 signaling involves activation of SRC‐like tyrosine kinase and metalloproteinases leading to activation of the HB‐EFG which, in turn, activates the EGFR signaling pathway leading to cell growth in response to the phosphorylation of Erk1/2 kinases.^74^, ^75^

The massive changes of subcellular proteins observed in the work here suggests numerous pathways involved in 4‐OHT response of MCF‐7 including some of which may explain the agonistic and antagonistic role of the drug. This dual behavior observed here demonstrates that in a complex cellular system the “real” response to 4‐OHT follows more complicated ways than those suggested and recorded by monitoring of single proteins. Thus, the study of pathways or metabolic processes of the most significantly changed proteins by quantitative subcellular proteomics is of relevant importance in revealing molecular basis of the Tam function.

In conclusion, the response of MCF‐7 cells to the Tam treatment shows significant changes in subcellular abundance rather than in their total abundance. Our data show: a) extensive redistribution of the subcellular abundance as a consequence of 4‐OHT; b) the relevance of moonlighting proteins in this proteomics study; and c) the most relevant pathways associated with agonistic and antagonistic response of Tam in MCF‐7. The results indicate the involvement of Tam in cardiomyopathy and GPCRs pathways indicating possible protective role of Tam against cardiovascular diseases as well as its potential role in enhancing breast tissue proliferation. This study demonstrates that in a complex cellular system the “real” response to 4‐OHT follows more complicated ways than those suggested and recorded by monitoring of single proteins.

## Conflict of Interest

The authors declare no conflict of interest.

## Supporting information

Supporting Information

Supporting Information

Supporting Information

Supporting Information

Supporting Information
