# Supplementary material for: Moonlighting Proteins and Cardiopathy in the Spatial Response of MCF‐7 Breast Cancer Cells to Tamoxifen
Source: Proteomics Clin Appl. 2019 Jul 25;13(5):1900029. doi: 10.1002/prca.201900029 (PMC6771495; doi:10.1002/prca.201900029)
Supplement: Supplementary file 5 — Supporting Information [file PRCA-13-na-s004.docx]

**Supplementary material**

**Moonlighting Proteins and Cardiopathy in the Spatial Response of MCF-7 Breast Cancer Cells to Tamoxifen**

Abdulrab Ahmed M Alkhanjaf, Roberto Raggiaschi, Mark Crawford, Gabriella Pinto^1^ and

Jasminka Godovac-Zimmermann^1^*

^a^ Proteomics and Molecular Cell Dynamics, Division of Medicine, School of Life and Medical Sciences, UCL, Royal Free Campus, Rowland Hill Street NW3 2PF, United Kingdom

^1^ Joint last authors

*Corresponding author: Prof J. Godovac-Zimmermann, e-mail: j. [godovac-zimmermann@ucl.ac.uk](mailto:godovac-zimmermann@ucl.ac.uk)

**Figure 1: Distribution of SILAC ratios (5 versus 3) for proteins from the total lysate (S_t_), nuclear (S_n_), and cytoplasmic (S_c_) samples.**

(Upper)_All proteins with ≥5 ratio counts for the union of the three replicates for each sample type; (Bottom)_All proteins with ≥3 ratio counts for all replicates of each sample type.

**
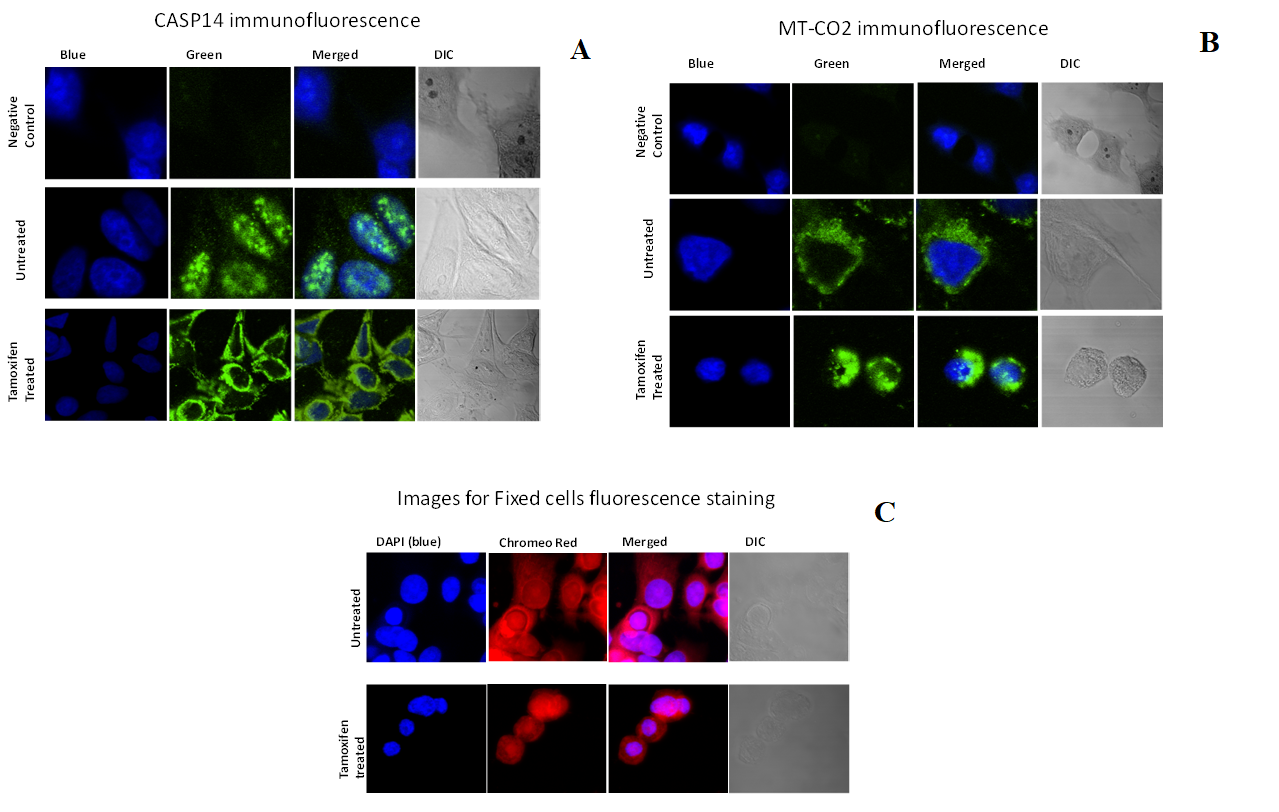
**

**Figure 2**. Immunofluorescence (IF) of the sub-cellular redistribution of CASPASE-14 protein (A) and of MT-CO2 protein (A) in MCF-7 cells.
